# Comparison of the total and hidden blood loss in patients undergoing single-level open and unilateral biportal endoscopic transforaminal lumbar interbody fusion: a retrospective case control study

**DOI:** 10.1186/s12891-023-06393-y

**Published:** 2023-04-14

**Authors:** Yu-Jian Peng, Zhi-Ying Fan, Qian-Liang Wang, Jun Dai, Qian-Zhong-Yi Zhang, Jun-Yin Cao, Xiao-Feng Liu, Jun Yan

**Affiliations:** Department of Orthopedic Surgery, The Second Affiliated Hospital of Soochow University, No.1055 Sanxiang Road, Gusu District, Suzhou, 215004 Jiangsu China

**Keywords:** Hidden blood loss, Total blood loss, Unilateral biportal endoscopy, Transforaminal lumbar interbody fusion, Degenerative lumbar disease

## Abstract

**Purpose:**

This study aimed to compare total blood loss (TBL) and hidden blood loss (HBL) in patients undergoing single-level open transforaminal lumbar interbody fusion (O-TLIF) and unilateral biportal endoscopic transforaminal lumbar interbody fusion (ULIF).

**Methods:**

A total of 53 patients who underwent ULIF and 53 patients who underwent O-TLIF from March 2020 to July 2022 were retrospectively reviewed. The Nadler’s formula was employed to estimate the patient’s blood volume (PBV), Gross’s formula to estimate TBL, and Sehat’s formula to estimate HBL. The obtained data were then analyzed with independent t test, chi-squared test, and analysis of covariance.

**Results:**

TBL and measured blood loss (MBL) in ULIF group (326.86 ± 223.45 ml, 99.00 ± 72.81 ml) was significantly lower than O-TLIF group (427.97 ± 280.52 ml, 270.66 ± 102.34 ml). Nevertheless, the HBL in ULIF group was higher than that in O-TLIF group (227.86 ± 221.75 ml vs 157.31 ± 268.08 ml), however this was not statistically significant (*p* = 0.143). The HBL was 69.71 ± 23.72% of TBL in ULIF group and 36.76 ± 18.79% of TBL in O-TLIF group. Patients in ULIF group had lower TBL and MBL, shorter duration of drainage, lower postoperative anemia, and shorter postoperative hospital stay compared to those in O-TLIF group.

**Conclusions:**

Perioperative HBL should not be neglected in patients undergoing ULIF or O-TILF, as it accounts for a large percentage of TBL in both groups. ULIF is associated with lower TBL and MBL, postoperative anemia, shorter postoperative hospital stays compared with O-TLIF.

**Supplementary Information:**

The online version contains supplementary material available at 10.1186/s12891-023-06393-y.

## Introduction

Posterior lumbar fusion is commonly used to treat degenerative spine disease [1]. Transforaminal lumbar interbody fusion (TLIF) is a surgical technique in which anterior column support is achieved using a posterolateral approach and unilateral cage insertion, along with posterior column stabilization using pedicle screw fixation to preserve posterior ligamentous structures [2, 3]. A literature review and meta-analysis showed that compared with posterior lumbar interbody fusion (PLIF), TLIF has fewer complications and shorter operation time [4]. However, conventional open TLIF (O-TLIF) is associated with significant soft tissue morbidity and a long recovery period, which may cause adverse outcomes [5, 6].

Numerous types of minimally invasive spine surgeries that minimize injury to normal anatomical structures have been proposed for treating lumbar degenerative disease [1, 2, 5, 7–9]. Relative to O-TLIF, minimally invasive TLIF (MI-TLIF) is reported to reduce intraoperative blood loss, postoperative pain, and time to discharge or recovery [10]. Recently, unilateral biportal endoscopic transforaminal lumbar interbody fusion (ULIF) has emerged as an alternative way of managing degenerative lumbar disease [9]. ULIF requires only two small incisions and further decreases muscle injury. Past studies indicate that compared to MI-TLIF, ULIF causes less early postoperative back pain, earlier ambulation, and shorter hospital stay [5].

Previous studies have attributed perioperative bleeding solely to measured blood loss (MBL) which includes intraoperative blood loss (IBL) and postoperative drainage volume. However, the actual total blood loss (TBL) is significantly greater than IBL and postoperative drainage only. Compared with the MBL, the hidden blood loss (HBL) is often ignored. The concept of HBL was first introduced by Sehat et al. [11] and its presence in orthopedic surgery was later confirmed by mounting evidence. Indeed, total blood loss (TBL) is composed of MBL and HBL. Hui Zhang et al. [12] reported that TBL was lower in MI-TLIF than in O-TLIF, while HBL was significantly higher in MI-TLIF. However, to the best of our knowledge, no studies have examined TBL and MBL in patients undergoing ULIF. Here, we compared TBL and MBL in patients undergoing single-level O-TLIF and ULIF.

## Patients and methods

### Patients

This retrospective study involved consecutive patients with single-level lumbar instability or degenerative disk disease who underwent O-TLIF or ULIF between March 2020 and October 2021. The inclusion criteria were, 1) patients with single operated segment O-TLIF or ULIF, 2) patients with unilateral neurological symptoms, unilateral decompressions and unilateral drainage, and 3) patients with complete clinical data. The exclusion criteria were: 1) previous lumbar surgery, 2) presence of infections and/or cancer, 3) patients on antiplatelet or anticoagulant medication, 4) patients with hematological malignancies, bleeding disorders, or chronic liver disease, 5) patients with missing data, and 6) patients who underwent autologous and allogeneic transfusion. A total of 106 patients met the inclusion criteria. Of these, 53 belonged to the ULIF group and 53 to the O-TLIF group. Two groups of surgery were completed by the same surgeon and surgical team. Ethical approval for this study was granted by the ethics committee of the Second Affiliated Hospital of Soochow University (No: JD-HG-2021–47). All methods were conducted in accordance with the ethical standards of the declaration of Helsinki. The following patient data were collected: gender, age, weight, height, body mass index (BMI), level of fusion, fibrinogen level, American Society of Anesthesiologists (ASA) classification score, operative time, length of postoperative hospital stay, preoperative and postoperative hematocrit (Hct), hemoglobin (Hb), and red blood cells (RBC).

### Surgical procedure

#### ULIF

**Fig. 1:**
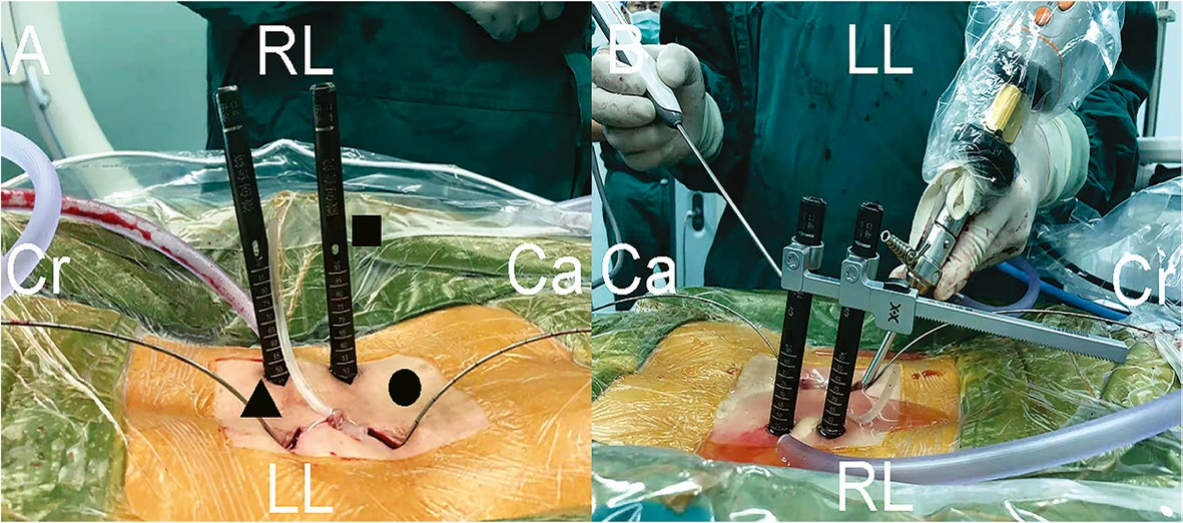
Photographs of the intraoperative scene. **A** Arrangement of the incisions. ▲A viewing portal.●A working portal. ■Percutaneous pedicle screw. **B** Intraoperative view of biportal endoscopic spinal surgery

**Fig. 2:**
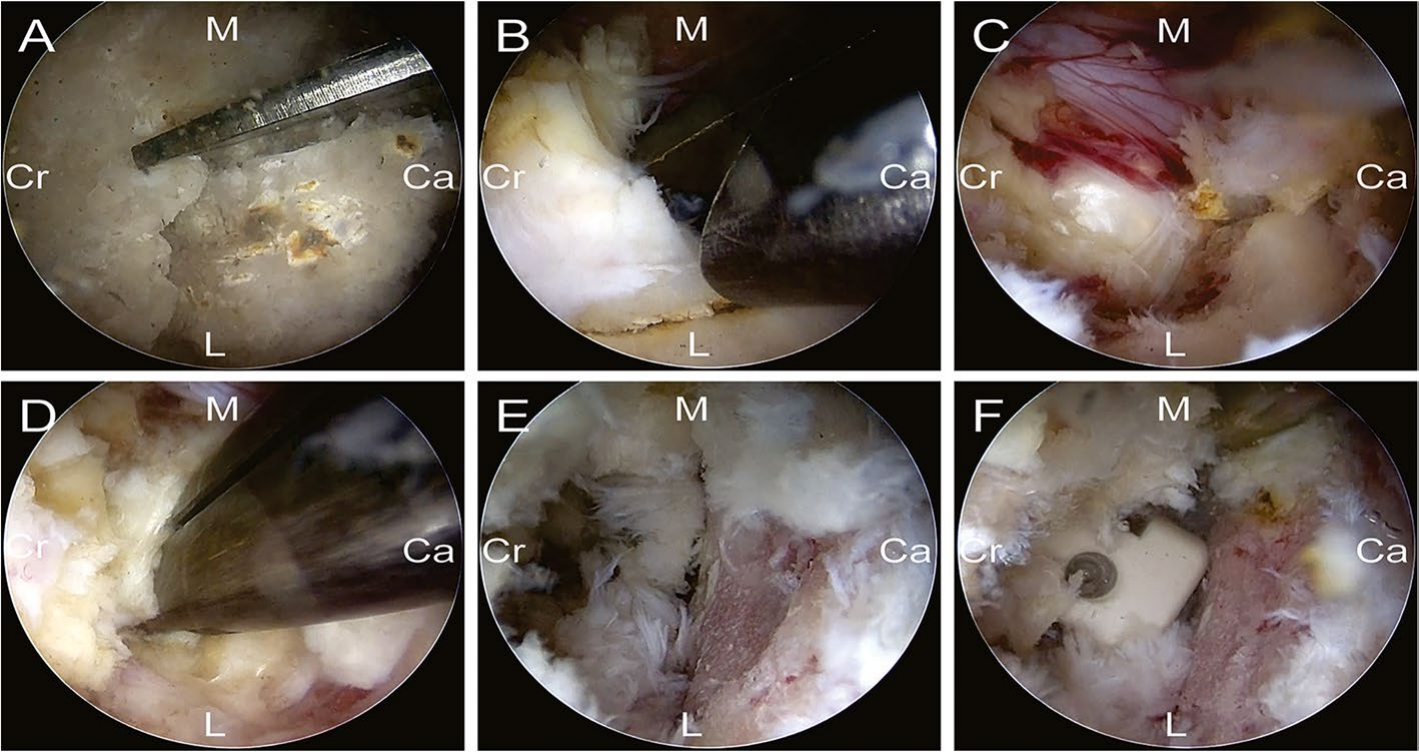
Images of the surgical procedure. **A** An ultrasonic bone scalpel was used for laminectomy and facetectomy. **B** Flavum ligament was removed by using forceps. **C** Exposure of disc space. **D** Pituitary forceps were used for removal of the intervertebral disc material. **E** Exposure of the disc space. **F** Endplate preparation. **G** A cage was filled with bone chips and inserted into the central part of the disc space

Taking the left-side approach as an example, patients were placed in prone position under general anesthesia. After intraoperative fluoroscopy, landmarks for skin incision were place 2 cm above and below the target intervertebral disc on the bilateral side central surface projection of the pedicle axis. Subsequently, Four skin incisions, about 1.5 cm long, were made both on the bilateral side according to the landmarks. Contralateral percutaneous pedicle screws were placed under fluoroscopic guidance via contralateral two incisions. The ipsilateral cranial portal was used as a viewing portal and the ipsilateral caudal portal was used as a working portal (Fig. 1A). Intraoperative view of biportal endoscopic spinal surgery was shown in Fig. 1B. Muscle and soft tissue were detached from the left proximal lamina to create workspace. Using an ultrasonic scalpel, ipsilateral laminectomy and facetectomy was performed (Fig. 2A), and the autologous bone harvested during these procedures used as bone grafts. The ligamentum flavum was then resected (Fig. 2B) and herniated disk was exposed (Fig. 2C). Pituitary forceps were then used to remove the intervertebral disc material (Fig. 2D). Endplate preparation was wholly done under endoscopic view (Fig. 2E). Using a bone grafting funnel, autologous bone were inserted and impacted into the intervertebral disc space. Next, using endoscopic guidance, a cage filled with local morselized bone was inserted (Fig. 2F). After cage insertion, the ipsilateral percutaneous pedicle screws were inserted through the viewing and working portals. Ipsilateral percutaneous pedicle screws and rods were then placed through the same incision. A negative pressure drainage tube was placed in the decompression side intraoperatively.

#### O-TLIF

The patient was placed in the prone position after general anesthesia. Then mark the target level under the C-arm guidance. Subperiosteal dissection was carried out to the tips of the spinous processes to expose the entry points for the pedicle screws. Pedicle screws were placed into the upper and subjacent vertebral pedicle of the segmental lesions. Facet joint and lamina were exposed. After unilateral laminectomy and inferior facetectomy, discectomy was done by retracting the traversing nerve root and dura medially. A cage fifilled with autologous bone was inserted in the disc space. The wound was copiously irrigated and closed in layers. A negative pressure drainage tube was placed in the decompression side intraoperatively.

### Perioperative fluid management strategy

Both groups adopted the same fluid management strategy. Perioperative fluid management strategy involved two strategies. First, all patients were given intravenous tranexamic acid 1.0 g at the start of the surgical procedure. Secondly, multiple drugs including antibiotics, non-steroidal analgesics, diuretics and proton pump inhibitor are used in the perioperative period. Total fluid infusion volume was 1000 mL approximately.

### Management of blood loss

No patient required blood transfusion during or after the operation. Complete blood counts were done on all patients including Hct, RBC, and Hb before surgery and on the second or third postoperative day. By this time, the patients were hemodynamically stable and fluid shifts would have been largely completed [13, 14].

Patients’ height and weight were recorded preoperatively. IBL was recorded by the anesthetist and included blood in suction bottles, as well as blood in weighed sponges used during the procedure. Postoperative drainage volume was recorded every 24 h and the drainage tube removed when the drainage volume in the surgical area was ≤ 50 mL/d.

### Patients’ blood volume

TBL was estimated by first determining patient blood volume (PBV) in milliliters using the following formula by Nadler et al. [15].

$$\mathrm{PBV }\left(\mathrm{mL}\right)=\left[{k1\times \mathrm{height}\left(m\right)}^{3}+k2\times \mathrm{weight}\left(kg\right)+k3\right]\times 1000.$$

For men: k1 = 0.3669, k2 = 0.03219, and k3 = 0.6041

For women: k1 = 0.3561, k2 = 0.03308, and k3 = 0.1833.

### Total blood loss

TBL was given by the product of PBV and Hct change using the following formula by Gross et al. [16]:

$$TBL\left(ml\right)=PBV\left(mL\right)\times \left({Hct}_{Pre}-{Hct}_{Post}\right)/{Hct}_{ave}.$$

where Hct_Pre_ is preoperative Hct, Hct_Post_ is 2^nd^ or 3^rd^ day postoperative Hct, and Hct_ave_ is the mean of Hct_Pre_ and Hct_Post_.

### Measured blood loss

MBL was calculated using the following formula:

$$MBL\;(mL)=Intraoperative\;blood\;loss\;(IBL,\;in\;mL)+Total\;postoperative\;drainage\;volume\;of\;the\;second\;or\;third\;postoperative\;day(mL).$$

IBL of O-TLIF was estimated by the volume of suction and the weight of gauze. Blood loss in gauze pieces was calculated by subtracting weight of dry gauze from the weight of blood soaked gauze pieces [17–19]. However, different from O-TLIF, continuous isotonic saline flow was maintained to provide a clear operative visual field in ULIF. Irrigation and suction were done simultaneously. Intraoperative hemorrhage was mixed with intraoperative irrigation. Because low IBL was detected intraoperatively but could not be calculated, the IBL in ULIF was disregarded. It should be emphasized that total postoperative drainage volume of the second or third postoperative day was not equal to TDV because extubation was not performed on the second or third postoperative day for all patients.

### Hidden blood loss

HBL was calculated using the following formula by Sehat et al. [13]:

$$HBL\;(mL)=TBL\;(mL)-MBL\;(mL).$$

### Anemia measurements

Anemia was indicated by a serum hemoglobin level < 130 g/L for men and < 120 g/L for women based on the World Health Organization’s criteria.

### Statistical analysis

All statistical analyses were done on SPSS version 20.0 (IBM). Values are presented as means ± SD. Continuous variables were compared using the independent t-test. Statistical significance of differences between categorical variables was tested using Chi-square test. The statistical significance of differences between groups was tested using univariate general linear model analysis of covariance. *P* < 0.05 was considered statistically significant.

## Results

Demographic data for the 58 patients are shown on Table 1. There was no significant difference between groups with regards to age, sex, weight, height, BMI, preoperative diagnosis, ASA classification, fibrinogen level, and the level of fusion between the 2 groups.

**Table 1:** Patients’ demographic information

Variable	ULIF	O-TLIF	*P*-value
Number of patients	53	53	
Gender (Male/Female)	23/30	30/23	0.174
Age (year)	54.79 ± 8.54	54.92 ± 12.03	0.948
Weight (kg)	64.87 ± 11.52	65.24 ± 17.21	0.897
Height (m)	1.63 ± 0.073	1.65 ± 0.096	0.249
BMI (kg/m2)	24.19 ± 3.30	23.74 ± 4.92	0.578
Preoperative diagnosis			
Lumbar spinal stenosis	47	51	0.141
Spondylolisthesis	6	2	
Level of fusion			
L2-L3	1	0	0.795
L3-L4	3	3	
L4-L5	29	29	
L5-S1	20	21	
ASA classification			
I	35	29	0.233
II	18	24	
Fibrinogen level	2.86 ± 0.58	2.83 ± 0.59	0.813
Patient’s blood volume (ml)	4008.11 ± 626.59	4092.18 ± 833.09	0.558
Preoperative haematocrit (%)	39.49 ± 4.44	40.85 ± 4.26	0.110
Preoperative hemoglobin (g/L)	133.02 ± 18.14	138.04 ± 15.56	0.129
Preoperative red blood cells (10^12/L)	4.48 ± 0.45	4.49 ± 0.48	0.913
Incidence of pre-operative anemia	24.53%	20.75%	0.643

Open transforaminal lumbar interbody fusion *O-TLIF*, Unilateral biportal endoscopic transforaminal lumbar interbody fusion *ULIF*, *BMI* Body mass index, American Society of Anesthesiologists *ASA*. Data are mean ± standard deviation; *P < 0.05

Relative to the O-TLIF group, ULIF was associated with significantly longer operation time and significantly shorter postoperative hospital stay (*p* = 0.000 and 0.042, respectively, Table 2). The ULIF group had shorter drainage duration (*p* = 0.003) and lower TDV (*p* = 0.000). Relative to the O-TLIF group, drainage volumes in the ULIF group were significantly lower in the first, second, and third postoperative day (*p* = 0.000, 0.000, and 0.000, respectively). Because the presence of physiological saline during ULIF made IBL difficult to calculate, ULIF-associated IBL was disregarded. The IBL in the O-TLIF group was 85.38 ± 23.20 mL. MBL results are shown in Table 3.

**Table 2:** Operative time and postoperative hospital stay information

Variable	ULIF	O-TLIF	*P*-value
Operative time (minute)	176.32 ± 32.89	130.87 ± 24.54	0.000*
Postoperative hospital stay (day)	5.51 ± 1.51	6.55 ± 3.31	0.042*

Open transforaminal lumbar interbody fusion *O-TLIF*, Unilateral biportal endoscopic transforaminal lumbar interbody fusion *ULIF*

Data are mean ± standard deviation; *P < 0.05

**Table 3:** The results of measured blood loss information

Variable	ULIF	O-TLIF	*P*-value
Duration of drainage (day)	2.21 ± 0.41	2.64 ± 0.92	0.003*
Total amount of postoperative drainage (ml)	97.09 ± 73.30	219.06 ± 140.13	0.000*
Drainage of first postoperative day (ml)	57.08 ± 39.61	124.91 ± 71.92	0.000*
Drainage of second postoperative day (ml)	33.79 ± 31.67	61.40 ± 35.30	0.000*
Drainage of third postoperative day (ml)	30.00 ± 16.73	49.04 ± 25.30	0.029*
Intraoperative blood loss (ml)	/	85.38 ± 23.20	/

Open transforaminal lumbar interbody fusion *O-TLIF*, Unilateral biportal endoscopic transforaminal lumbar interbody fusion *ULIF*

Data are mean ± standard deviation; *P < 0.05

**Fig. 3:**
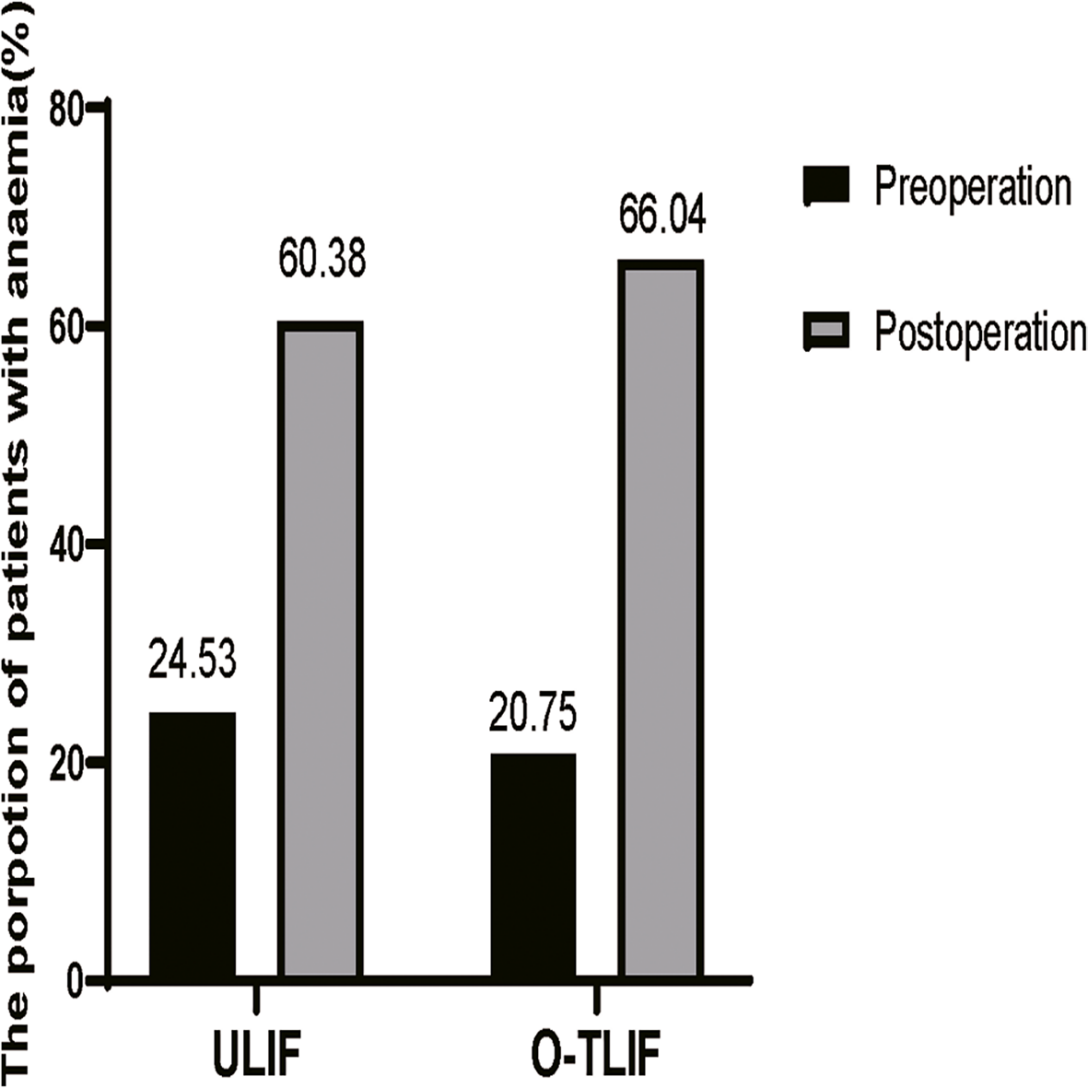
The propotion of patients with anaemia in 2 groups. Open transforaminal lumbar interbody fusion, O-TLIF; Unilateral biportal endoscopic transforaminal lumbar interbody fusion, ULIF

**Fig. 4:**
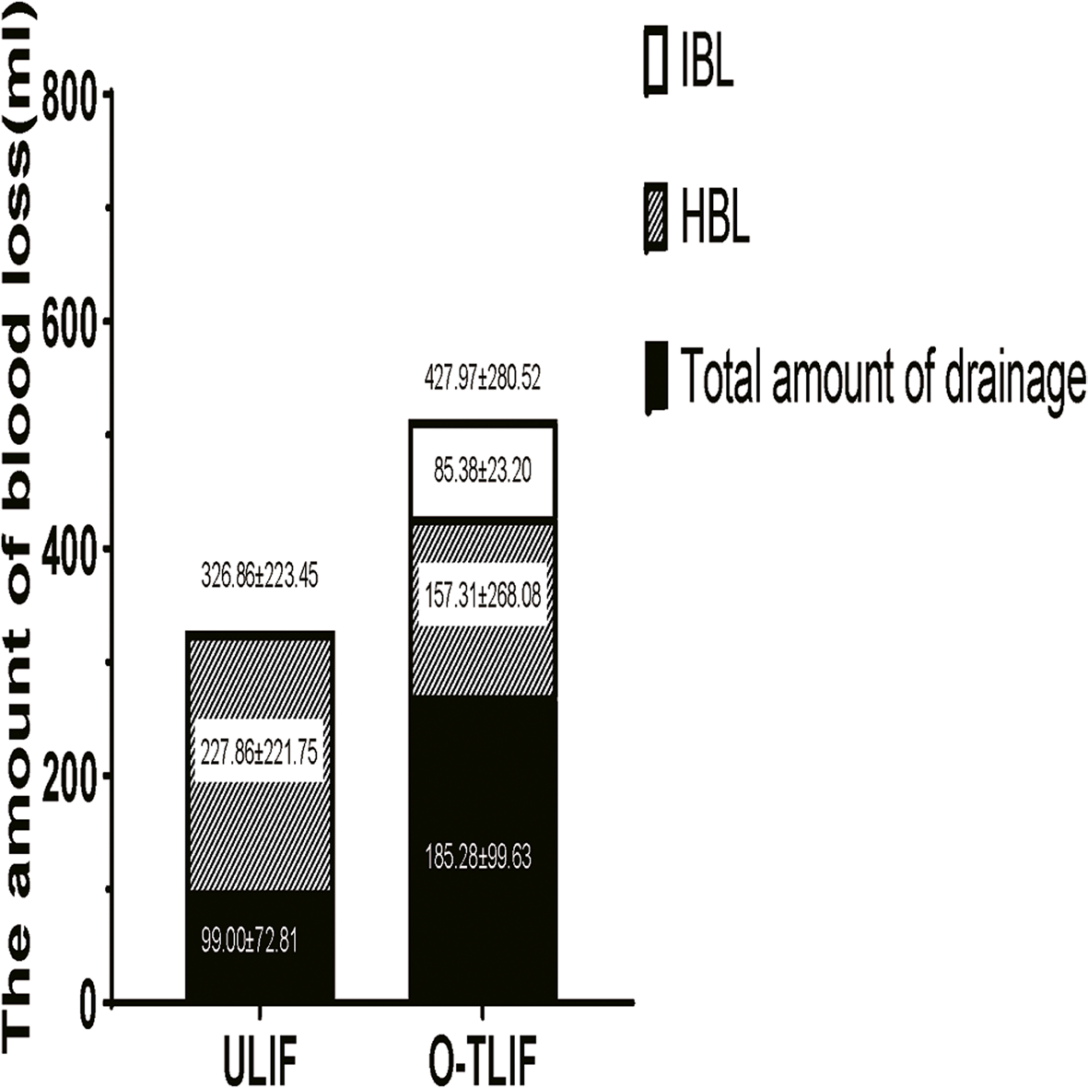
The composition of total blood loss in 2 groups. Open transforaminal lumbar interbody fusion, O-TLIF; Unilateral biportal endoscopic transforaminal lumbar interbody fusion, ULIF; Intraoperative blood loss, IBL; Hidden blood loss, HBL

Analysis of perioperative blood revealed that relative to the O-TILF group, the ULIF group had significantly higher levels of postoperative Hct, Hb, and RBC (*p* = 0.000, 0.000, and 0.000, respectively; Table 4). The 2 groups did not differ significantly with regards to PBV and HBL. However, relative to the O-TLIF group, the HBL of TBL was significantly higher in the ULIF group (36.76 ± 18.79% vs 69.71 ± 23.72%, *p* = 0.010). MBL was significantly higher in the O-TILF group (270.66 ± 102.34 mL) than in the ULIF group (99.00 ± 72.81 mL). The TBL was 427.97 ± 280.52 ml (10.45 ± 5.87% of PBV) in O-TLIF group and 326.86 ± 223.45 ml (8.15 ± 5.37% of PBV) in ULIF group. TBL were significantly higher in the O-TILF group than in the ULIF group (*p* = 0.000). The proportions of patients with anemia in the 2 groups are shown in Fig. 3. The composition of total blood loss in the 2 groups is shown in Fig. 4.

**Table 4:** The result of perioperative blood changes in patient

Variable	ULIF	O-TLIF	*P-value*
Preoperative haematocrit (%)	39.49 ± 4.43	40.85 ± 4.25	0.000*
Postoperative haematocrit (%)	36.37 ± 4.24	36.64 ± 4.42	
Preoperative hemoglobin (g/L)	133.02 ± 18.14	138.04 ± 15.56	0.000*
Postoperative hemoglobin (g/L)	119.07 ± 15.70	120.23 ± 15.40	
Preoperative red blood cells (10^12/L)	4.48 ± 0.45	4.49 ± 0.48	0.000*
Postoperative red blood cells (10^12/L)	4.05 ± 0.38	3.96 ± 0.51	
Patient’s blood volume (ml)	4008.11 ± 626.59	4092.18 ± 833.09	0.558
Total blood loss (ml)	326.86 ± 223.45	427.97 ± 280.52	0.043*
Measured blood loss (ml)	99.00 ± 72.81	270.66 ± 102.34	0.000*
Hidden blood loss (ml)	227.86 ± 221.75	157.31 ± 268.08	0.143
TBL as a % of PBV	8.15 ± 5.37	10.45 ± 5.87	0.000*
HBL as a % of TBL	69.71 ± 23.72	36.76 ± 18.79	0.000*

Open transforaminal lumbar interbody fusion *O-TLIF*, Unilateral biportal endoscopic transforaminal lumbar interbody fusion *ULIF*, Total blood loss *TBL*, Hidden blood loss *HBL*, Patient’s blood volume *PBV*. Data are mean ± standard deviation, *P < 0.05

## Discussion

Since its introduction in 2000, several studies have reported HBL in various types of orthopedic surgeries. Sehat et al. [11] found that mean HBL was 735 mL, accounting for 50% of TBL during total knee arthroplasty. Yoji Ogura et al. [20] showed that during 2- to 3-level posterior lumbar fusion, HBL varies from 678–1,267 mL. Importantly, Foss and Kehlet et al. [21] reported that HBL was consistently associated with in-hospital complications and extended length of hospital stay. But as for now, no studies have reported HBL in ULIF. Here, we performed a retrospective analysis of HBL and TBL during ULIF and O-TLIF.

Wang et al. [22] reported that the mean estimated blood loss was 126.03 ± 17.85 ml in ULIF surgery. However, ULIF’s IBL was minimal and difficult to calculate because of the volume of saline solution used for irrigation. Thus, in this study, the IBL of ULIF was neglected and incorporated into the calculations of HBL. HBL may result from blood hemolysis [23, 24], extravasation of blood into tissue compartments [25], and free fatty acids mediated oxidative damage of RBCs and Hb [26].

Previous studies [27–30] indicate that gender, multilevel, operative time, fibrinogen level, ASA classification, autologous and allogeneic transfusion, BMI, and surgical method were independent risk factors for HBL in posterior lumbar interbody fusion. Here, to investigate the effect of surgical method on HBL, we used other variables as control variables. Our data show that mean HBL was 227.86 ± 221.75 mL in ULIF (constituting up to 69.71% of TBL) and 157.31 ± 268.08 ml in O-TLIF (constituting up to 36.76% of TBL). Surprisingly, mean HBL did not differ significantly between the two groups. However, the proportion of HBL in the two groups was significantly different. Our data indicate that HBL level was considerable and that it was the most important contributor to TBL in both ULIF and TLIF, which is consistent with past findings [12, 31]. The difference in HBL composition between the groups may be explained by the following. 1) One non-negligible reason is that IBL in ULIF was neglected and factored into the HBL calculation. 2) The higher radiofrequency used in ULIF may have generated more oxidizing species that damaged RBCs and Hb. 3) HBL might be affected by the “learning curve” that accompanies the introduction of any new surgical method, resulting in higher HBL being observed during ULIF in the initial cases.

In our study, the MBL in O-TLIF (270.66 ± 102.34 mL) was significantly higher than in ULIF (99.00 ± 72.81 mL). ULIF involved two minimally invasive incisions; one for endoscopic viewing and the other for the insertion and manipulation of surgical tools. The advantages of ULIF include being minimally invasive, involving less muscular dissection, better surgical view, and more precise operation. These factors may explain the lower TBL in the ULIF group. Moreover, considering that ULIF was wholly performed in aqueous media, we pulled with all strength to hemostasis because even minor bleeding can obstruct the surgeon field of vision. Moreover, Xu et al. [32] pointed out that the components of drainage changed radically with time. Thus, rather than the drainage volume, the true blood component of the drainage should be taken into account. In contrast with O-TLIF, ULIF leaves a high volume of water in the muscle and spinal space following saline irrigation. From this, it is expected that there was more water in ULIF drainage. Thus, the true blood volume was less than the drainage volume we described and the true difference between the two groups may be larger.

Perioperative anemia is significantly associated with complications and length of hospital stay [33, 34]. TBL only accounted for 8.15 ± 5.37% of PBV in ULIF, which was markedly lower than in the O-TLIF group. As shown on Fig. 3, lower TBL reduced the incidence of postoperative anemia in the ULIF group. As expected, relative to O-TLIF, ULIF was associated with significantly shorter postoperative hospital stay. HBL is the leading cause of perioperative anemia and hidden blood loss and perioperative anemia may be minimized by various interventions. A recent study [35] found that tranexamic acid reduces HBL during posterior lumbar interbody fusion surgery. Hong Qian et al. [36] found out that antioxidants attenuate oxidative stress-induced HBL in rats. However, more research is needed to identify new strategies for reducing HBL.

This study has some limitations. First, being retrospective, this study is inevitably susceptible to bias. Secondly, the study’s sample size was relatively small. Thirdly, disregarding ULIF’s IBL may have influenced our findings. Finally, studies [37, 38] show that fluid shifts may not be completed in all patients in 2 or 3 days after the operation. Thus, the Hct_Post_ we used to calculate TBL may not precise.

## Conclusion

In summary, perioperative HBL should not be neglected when performing ULIF or O-TILF as it accounts for a large percentage of TBL in both groups. Lower TBL and MBL in the ULIF group lowers the incidence of postoperative anemia in patients, thereby reducing the length of postoperative hospital stay.

## Supplementary Information

**Additional file 1:** 

## Data Availability

The original contributions presented in the study are included in the article Supplementary Material, further inquiries can be directed to the corresponding author.

## Abbreviations

TBL: Total blood loss
HBL: Hidden blood loss
PBV: Patient’s blood volume
MBL: Measured blood loss
IBL: Intraoperative blood loss
BMI: Body mass index
ASA: American Society of Anesthesiologists
Hct: Hematocrit
Hb: Hemoglobin
RBC: Red blood cells
O-TLIF: Open transforaminal lumbar interbody fusion
ULIF: Unilateral biportal endoscopic transforaminal lumbar interbody fusion
TLIF: Transforaminal lumbar interbody fusion
PLIF: Posterior lumbar interbody fusion
MI-TLIF: Minimally invasive Transforaminal lumbar interbody fusion
